# Associations of e-cigarette experimentation with support for tobacco control policies in the European Union, 2012-2014

**DOI:** 10.18332/tid/84865

**Published:** 2018-03-16

**Authors:** Anthony A. Laverty, Filippos T. Filippidis, Esteve Fernandez, Constantine I. Vardavas

**Affiliations:** 1 Public Health Policy Evaluation Unit, School of Public Health, Imperial College London, London, United Kingdom; 2 Department of Primary Care and Public Health, School of Public Health, Imperial College London, London, United Kingdom; 3 Tobacco Control Unit, WHO Collaborating Center for Tobacco Control, Institut Català d’Oncologia-ICO, Barcelona, Spain; 4 Clinic of Social and Family Medicine, School of Medicine, University of Crete, Heraklion, Greece

**Keywords:** smoking, non-cigarette tobacco products, tobacco, smoking cessation, public opinion

## Abstract

**INTRODUCTION:**

There are limited data on the potential effects of e-cigarette experimentation on support for tobacco control policies. To bridge this gap, we assessed associations between e-cigarette experimentation and support for tobacco control policies in the European Union 2012-2014. We also investigated variations across tobacco-use status, e-cigarette experimentation and socio-demographic characteristics.

**METHODS:**

Datasets were used from the Special Eurobarometer for Tobacco surveys performed in 2012 (n=26 751) and 2014 (n=27 801). Tobacco control policies assessed were: banning advertising, policies to keep tobacco out of sight, banning online sales, banning flavors, standardized packaging, tax increases, and policies to reduce illicit trade in tobacco. We use multilevel logistic regression models to assess variations in socio-demographics and tobacco/e-cigarette use with support for these policies in 2014, and examined changes in support for these policies, between 2012 and 2014, separately by tobacco-use status (never, current, and former smokers).

**RESULTS:**

Population support for tobacco control policies was high in 2014: policies to reduce illicit trade had the highest level of support at 70.1%, while tax increases were the least likely measure to be supported with 52.3% support. Among never and former smokers, experimentation with e-cigarettes was associated with reduced support for all tobacco control policies assessed. For example, never smokers who had experimented with e-cigarettes were less likely to support either tobacco advertising bans (adjusted odds ratio aOR=0.57, 95% confidence interval 0.46-0.71) or standardized packaging for tobacco (aOR=0.58, 95% CI: 0.47-0.71). Former smokers who had experimented with e-cigarettes were less likely to either support standardized packaging for tobacco (aOR=0.70, 95% CI: 0.60-0.82) or keeping tobacco out of sight (aOR=0.77, 95% CI: 0.65-0.90). Among current smokers, e-cigarette experimentation was not associated with support for the tobacco control policies assessed.

**CONCLUSIONS:**

E-cigarette experimentation was consistently associated with reduced support for tobacco control policies among never and former smokers but not among current smokers. The implications of these findings for tobacco control are unknown, but the data support concerns that e-cigarette experimentation may affect public support for established tobacco control policies within specific subgroups. Further research is needed to assess potential long-term impacts on tobacco control policies.

## INTRODUCTION

The increase in e-cigarette use in Europe has introduced questions about its impact on population health, as well as potential effects on support for tobacco control policies^1,2^. The recent implementation of the Tobacco Products Directive (TPD) in the European Union (EU) and transposition into specific legislations in most EU Member States (EU MS) has set the stage to standardize elements of tobacco and e-cigarette product design and regulation across the EU MS^3^. While the TPD provides an outline of actions to be handled centrally, it still leaves many details of regulation of both tobacco products and e-cigarettes within the responsibility of the EU MS. Specifically for e-cigarettes, the TPD only applies to e-cigarettes that contain nicotine while individual Member States can decide how to regulate products that do not contain nicotine.

Public support for tobacco control policies in the past has been important in both the passing of any legislation and its enforcement^4^. There are variations in public support for many policies across locations, as well as differences in how these issues are regulated^5-8^. The proliferation of e-cigarettes has led several countries to review and amend their tobacco control legislation; the debate about these issues is expected to intensify in upcoming years^9^. In the EU there has been an increase in the percentage of the population having ever tried e-cigarettes from 7% in 2012 to 12% in 2014, with high levels of variation in use between individual Member States^2^. Nevertheless, potential impacts on public support for tobacco control policies are yet to be analysed in detail. Nationally representative and comparable data between EU MS could assist in supporting any national legislative actions but will also contribute to our understanding of the impact that e-cigarettes may have on tobacco control legislations in general, in light of concerns regarding the renormalization of smoking^1,10^. To address this evidence gap, the aims of this study were to investigate changes in support for tobacco control policies in the EU between 2012 and 2014, and to investigate the role of e-cigarette experimentation on support for these policies. Our hypothesis was that support for the tobacco control policies would be lower amongst people who had experimented with e-cigarettes, and that overall support for these policies would have declined between 2012 and 2014.

## METHODS

### Data source

We conducted secondary analyses of data from waves 77.1 (2012) and 82.4 (2014) of the Special Eurobarometer on Tobacco survey collected in 27 and 28 EU Member States, respectively^11,12^. A multistage sampling design was employed in each wave to collect samples representative of the population aged ≥15 years, both at EU and EU MS level. Face-to-face interviews were used to record self-reported data on tobacco use and socio-demographic characteristics; all interviews were conducted at participants’ homes and in the local language. Post-stratification and population-size weighting were applied based on official Eurostat data on gender, age and area of residence, to ensure samples were representative. Croatia was excluded from the 2012-2014 analyses as it was not a member of the EU in 2012 and hence not in that year’s dataset. We used a repeated cross-sectional design and the total sample of participants was 26 751 in 2012 and 27 801 in 2014 (26 792 excluding Croatia). Descriptive results of the surveys are presented in the official Eurobarometer reports^13,14^.

### Policies

#### Support for tobacco control policies

All participants were asked to state if they would be in favour or opposed (or ‘don’t know’) to each of the following tobacco control policies: a) banning advertising of tobacco products in shops or points of sales, b) increasing taxes on tobacco products, c) banning colours, logos and promotional elements from tobacco-product packaging (product design features), d) banning flavors that make tobacco products more attractive, e) keeping tobacco products out of sight in shops or points of sale, f) improving the traceability of tobacco products in order to reduce their illicit trade, even if this makes them a few cents more expensive (reducing illicit trade), g) banning the sales of tobacco via the Internet (Internet sale). For the purpose of our analyses, those opposed and those who responded ‘don’t know’ were classified as ‘not in favour’. We have used this classification to focus on specific support for policies, and thus have combined these two groups under this rationale. Weighted percentages of respondents stating ‘don’t know’ if they support policies ranged from 8.3% for raising taxes to 13.8% for standardized packaging.

#### Tobacco smoking

All participants were asked ‘Regarding smoking cigarettes, cigars or a pipe, which of the following applies to you?’. Response choices were ‘You currently smoke’ (i.e. current smokers), ‘You used to smoke but you have stopped’ (i.e. former smokers), and ‘You have never smoked’ (i.e. never smokers).

#### E-cigarette use

For 2014 e-cigarette use was assessed with the question ‘Regarding the use of electronic cigarettes or any similar electronic devices (eshisha, e-pipe), which of the following statements applies to you?’ with response choices: ‘You currently use electronic cigarettes or similar electronic devices (e.g. e-shisha, e-pipe)’; ‘You used them in the past, but no longer use them’; ‘You tried them in the past but no longer use them’; ‘You have never used them’; and ‘Don’t know’. Any one of the first three responses was considered experimentation with e-cigarettes. For 2012 e-cigarette use was assessed with the question ‘Have you ever tried any of the following products? Electronic cigarettes’, and response choices included: ‘Yes, you use or used it regularly’; ‘Yes, you use or used it occasionally’; ‘Yes, you tried it once or twice’; ‘No’; and ‘Don’t know’. Respondents who reported that they had ever used or tried e-cigarettes were classified as having experimented with e-cigarettes.

#### Socio-demographic data

The surveys also collected data on participants’ age (15-24, 25-39, 40-54, and ≥55 years), gender (male, female), age when they stopped full-time education (≤15, 16-19 and ≥20 years), area of residence (rural, urban), and their difficulties to pay bills during the last twelve months (almost never/never, and from time to time/most of the time) as a proxy of socioeconomic status.

### Statistical analysis

Descriptive results are presented as percentages with 95% confidence intervals (CI) and logistic regression models are presented as adjusted odds ratios (aOR) with 95% CI. All descriptive results have been obtained with the use of weights provided in the Eurobarometer dataset to account for the complex design of the survey. We also compared unadjusted proportions of support for individual policies using chi-squared tests.

To assess the associations of e-cigarette experimentation and tobacco use with support for each of the aforementioned tobacco control policies in 2014, we used multilevel logistic regression, which allows for clustering within each EU MS, with level 1 being individuals and level 2 EU MS. We used likelihood ratio tests to compare single and multilevel models, as well as to decide on the optimal specification of the regression models. We created a combined variable to account for both e-cigarette ever use and tobacco use, with those never smoking tobacco or using e-cigarettes as the reference category. To assess associations of e-cigarette experimentation and survey year with support for each of these policies, we performed analyses stratified by tobacco-use status (never smokers, former smokers and current smokers). All models were adjusted for age, gender, area of residence, education, and difficulty to pay bills, performed with Stata 14.0.

## RESULTS

### Trends in support for tobacco control policies in the EU

For 2012, 50.7% of the sample participants were never smokers, 27.9% were current smokers and 21.4% were former smokers (Table 1). For 2014, these percentages were 54.3%, 26.2% and 19.5%, respectively.

**Table 1:** Characteristics of the respondents of the Special Eurobarometer on Tobacco sample in 2012 (n=26 751) and 2014 (n=26 792)^1^

	*2012 N (weighted %)*	*2014 N (weighted %)*
**Gender**		
Female	14 466 (51.7)	14 687 (51.6)
Male	12 285 (48.3)	12 105 (48.4)
**Age (years)**		
≥55	10 635 (35.1)	11 851 (36.6)
40-54	6 946 (25.9)	6 770 (26.0)
25-39	6 190 (24.7)	5 565 (23.9)
18-24	2 980 (14.4)	2 606 (13.5)
**Education (age at completion)**		
≤15	4 867 (21.1)	4 532 (18.8)
16-19	12 656 (47.6)	12 041 (47.3)
≥20	8 751 (31.2)	9 777 (33.9)
**Area of residence**		
Rural	9 546 (34.0)	8 099 (29.0)
Urban	17 176 (66.0)	18 679 (71.0)
**Difficulty in paying bills**		
Almost never/never	15 785 (63.8)	17 096 (66.4)
From time to time/most of the time	10 448 (36.2)	9 306 (33.6)
**Smoking status**		
Never smoker	13 550 (50.7)	14 619 (54.3)
Current smoker	7 352 (27.9)	6 516 (26.2)
Former smoker	5 782 (21.4)	5 589 (19.5)
		

^1^Excluding Croatia from the 2014 data set.

Policies to reduce illicit trade were the most likely policy to be supported in both 2012 (73.5%) and 2014 (70.1%) (Table 2). Tax increases were the least likely measure to be supported in both years, 53.2% in 2012 and 52.3% in 2014, while 64.0% of participants supported advertising bans in 2012 and 66.9% in 2014. Support for standardized packaging was 56.7% in 2012 and 55.3% in 2014.

**Table 2:** Support for selected tobacco control measures in 27 EU Member States, 2012-2014, among the respondents of the Special Eurobarometer on Tobacco sample in 2012 (n=26 751) and 2014 (n=26 792)^1^

	*2012 % (95% CI)*	*2014 % (95% CI)*	p*
Ban advertising	64.0 (63.1-64.9)	66.9 (66.0-67.8)	<0.001
Tobacco out of sight	58.2 (57.3-59.1)	59.7 (58.7-60.6)	<0.001
Ban online sales	62.3 (61.4-63.2)	63.7 (62.8-64.7)	<0.001
Ban flavors	63.0 (62.1-63.9)	61.9 (60.9-62.8)	0.942
Standardized packaging	56.7 (55.8-57.6)	55.3 (54.3-56.3)	0.406
Tax increases	53.2 (52.3-54.2)	52.3 (51.4-53.3)	0.006
Reduce illicit trade	73.5 (72.6-74.3)	70.1 (69.2-71.0)	<0.001

^1^Excluding Croatia from the 2014 data set.

* Chi-squared test

Gender, age, educational level and socioeconomic status were associated with support for specific tobacco control policies (Table 3). For instance, those more educated were more likely to support a ban on tobacco advertising, tobacco displays, tax increases and methods to reduce illicit trade. In contrast, men were less likely to compare a number of policies, as were younger respondents. People with difficulty in paying bills were less likely to support tax increases (aOR=0.88, 95% CI: 0.83-0.94) and policies to reduce illicit trade (aOR=0.84, 95% CI: 0.79-0.90).

**Table 3:** Variations in support by respondent characteristics and tobacco use, for tobacco control policies in 27 EU Member States, 2014 (n=26 792)

		*Ban advertising aOR (95% CI)*	*Tobacco out of sight aOR (95% CI)*	*Ban online sales aOR (95% CI)*	*Ban flavors aOR (95% CI)*	*Standardized packaging aOR (95% CI)*	*Tax increases aOR (95% CI)*	*Reduce illicit trade aOR (95% CI)*
**Gender**	Female	ref	ref	ref	ref	ref	ref	ref
	Male	0.89 (0.84-0.94)	0.84 (0.80-0.89)	0.86 (0.82-0.91)	0.89 (0.84-0.94)	0.86 (0.82-0.91)	0.97 (0.92-1.03)	0.97 (0.91-1.02)
**Age (years)**	≥55	ref	ref	ref	ref	ref	ref	ref
	40-54	0.94 (0.88-1.01)	0.90 (0.83-0.96)	0.89 (0.83-0.95)	0.84 (0.79-0.91)	0.87 (0.81-0.93)	0.93 (0.86-1.00)	0.97 (0.90-1.04)
	25-39	0.82 (0.76-0.89)	0.83 (0.77-0.90)	0.81 (0.75-0.87)	0.72 (0.67-0.77)	0.71 (0.66-0.77)	0.95 (0.88-1.03)	0.93 (0.85-1.00)
	18-24	0.63 (0.57-0.70)	0.64 (0.58-0.71)	0.63 (0.57-0.69)	0.51 (0.46-0.56)	0.53 (0.48-0.58)	0.77 (0.70-0.86)	0.80 (0.72-0.89)
**Education (age at completion)**	≤15	ref	ref	ref	ref	ref	ref	ref
	16-19	0.99 (0.91-1.08)	1.00 (0.92-1.08)	1.09 (1.00-1.18)	0.99 (0.91-1.07)	1.05 (0.97-1.14)	0.98 (0.91-1.07)	1.12 (1.02-1.22)
	≥20	1.13 (1.03-1.24)	1.10 (1.00-1.20)	1.06 (0.97-1.15)	1.00 (0.91-1.09)	1.05 (0.97-1.15)	1.12 (1.02-1.22)	1.30 (1.19-1.42)
**Area of residence**	Rural	ref	ref	ref	ref	ref	ref	ref
	Urban	0.98 (0.93-1.05)	0.96 (0.91-1.02)	1.01 (0.95-1.07)	0.98 (0.93-1.04)	0.96 (0.90-1.01)	1.00 (0.95-1.07)	1.05 (0.98-1.11)
**Difficulty paying bills**	Almost never/never	ref	ref	ref	ref	ref	ref	ref
	From time to time/most of the time	1.01 (0.94-1.07)	0.99 (0.93-1.05)	1.00 (0.94-1.07)	1.02 (0.95-1.08)	1.02 (0.96-1.09)	0.88 (0.83-0.94)	0.84 (0.79-0.90)
**Tobacco and e-cigarette use**	Never smoker/never used e-cigarette	ref	ref	ref	ref	ref	ref	ref
	Never smoker/have used e-cigarette	0.57 (0.43-0.74)	0.66 (0.51-0.86)	0.55 (0.43-0.72)	0.50 (0.39-0.65)	0.62 (0.48-0.80)	0.60 (0.47-0.78)	0.52 (0.40-0.68)
	Former smoker/never used e-cigarette	0.69 (0.64-0.75)	0.68 (0.63-0.73)	0.77 (0.71-0.83)	0.72 (0.67-0.78)	0.70 (0.65-0.75)	0.60 (0.47-0.78)	0.77 (0.71-0.83)
	Former smoker/have used e-cigarette	0.48 (0.40-0.58)	0.53 (0.43-0.67)	0.57 (0.47-0.69)	0.49 (0.41-0.60)	0.47 (0.39-0.57)	0.38 (0.31-0.46)	0.60 (0.49-0.73)
	Current smoker/never used e-cigarette	0.30 (0.28-0.32)	0.29 (0.27-0.31)	0.36 (0.34-0.39)	0.31 (0.28-0.33)	0.29 (0.27-0.31)	0.10 (0.09-0.10)	0.31 (0.28-0.33)
	Current smoker/have used e-cigarette	0.34 (0.31-0.38)	0.35 (0.31-0.39)	0.37 (0.33-0.41)	0.30 (0.27-0.33)	0.30 (0.27-0.33)	0.10 (0.09-0.11)	0.34 (0.31-0.38)

AOR: Adjusted Odds Ratio. Adjusted for age; gender; area of residence; education; difficulty to pay bills and all the other variables shown in the table in multilevel logistic regression models with country used as the higher level of analysis.

Ever use of both e-cigarettes and tobacco were consistently associated with reduced support for tobacco control policies assessed. Compared with those who have never smoked tobacco or used an e-cigarette, never smokers who had used e-cigarettes were less likely to support all policies, including banning flavors (aOR=0.50, 95% CI: 0.39-0.65) and measures to reduce illicit trade (aOR=0.52, 95% CI: 0.40-0.68). Former smokers who had used e-cigarettes were less likely to support all policies including tax increases (aOR=0.38, 95% CI: 0.31-0.46). Current smokers who had never used e-cigarettes were less likely to support tax increases (aOR=0.10, 95% CI: 0.09-0.10), while current smokers who had used e-cigarettes were less likely to support measures, including standardized packaging (aOR=0.30, 95% CI: 0.27-0.33) and banning flavors (aOR=0.30, 95% CI: 0.27-0.33).

Among never smokers, support for advertising bans (aOR=1.15, 95% CI: 1.09-1.22), keeping tobacco out of sight (aOR=1.12, 95% CI: 1.06-1.18) and banning online sales (aOR=1.09, 95% CI: (1.03-1.15) increased between 2012 and 2014, while support for policies to reduce illicit trade decreased (aOR=0.84, 95% CI: 0.79-0.89) (Table 4). Among never smokers, those who reported e-cigarette experimentation were less likely to support all tobacco control policies assessed in the Eurobarometer.

**Table 4:** Stratified analyses of the impact of tobacco use and e-cigarette experimentation on support for tobacco control policies in 27 EU Member States, 2012 -2014

		*Ban advertising aOR (95% CI)*	*Tobacco out of sight aOR (95% CI)*	*Ban online sales aOR (95% CI)*	*Ban flavors aOR (95% CI)*	*Standardized packaging aOR (95% CI)*	*Tax increases aOR (95% CI)*	*Reduce illicit trade aOR (95% CI)*
**Among never smokers (N=28 169)**								
Year	2012 (ref)	ref	ref	ref	ref	ref	ref	ref
	2014	1.15 (1.09-1.22)	1.12 (1.06-1.18)	1.09 (1.03-1.15)	1.00 (0.94-1.05)	0.96 (0.91-1.01)	0.96 (0.91-1.01)	0.84 (0.79-0.89)
**E-cigarette experimentation**	No (ref)	ref	ref	ref	ref	ref	ref	ref
	Yes	0.57 (0.46-0.71)	0.66 (0.53-0.82)	0.55 (0.44-0.68)	0.48 (0.39-0.59)	0.58 (0.47-0.71)	0.59 (0.47-0.72)	0.48 (0.38-0.59)
**Among current smokers (N=13 868)**								
Year	2012 (ref)	ref	ref	ref	ref	ref	ref	ref
	2014	1.03 (0.96-1.11)	1.01 (0.94-1.09)	1.03 (0.96-1.11)	0.91 (0.85-0.98)	0.90 (0.84-0.97)	1.06 (0.97-1.16)	0.78 (0.73-0.84)
**E-cigarette experimentation**	No (ref)	ref	ref	ref	ref	ref	ref	ref
	Yes	1.03 (0.94-1.12)	1.09 (1.00-1.18)	0.95 (0.87-1.03)	0.94 (0.86-1.02)	1.01 (0.93-1.10)	1.02 (0.92-1.14)	1.07 (0.98-1.16)
**Among former smokers (N=11 371)**								
Year	2012 (ref)	ref	ref	ref	ref	ref	ref	ref
	2014	1.10 (1.01-1.19)	1.09 (1.00-1.18)	1.16 (1.07-1.25)	1.04 (0.96-1.12)	1.00 (0.92-1.08)	0.93 (0.86-1.01)	0.90 (0.82-0.98)
**E-cigarette experimentation**	No (ref)	ref	ref	ref	ref	ref	ref	ref
	Yes	0.73 (0.62-0.86)	0.77 (0.65-0.90)	0.72 (0.61-0.84)	0.72 (0.61-0.84)	0.70 (0.60-0.82)	0.62 (0.53-0.72)	0.71 (0.60-0.84)

AOR: Adjusted Odds Ratio. Adjusted for age; gender; area of residence; education; difficulty to pay bills and all the other variables shown in the table in multilevel logistic regression models with country used as the higher level of analysis.

Among current smokers there were no increases in support for tobacco control policies between 2012 and 2014 but a decrease in support for banning flavors (aOR=0.91, 95% CI: 0.85-0.98), standardized packaging (aOR=0.90, 95% CI: 0.84-0.97) and policies to reduce illicit trade (aOR=0.78, 95% CI: 0.73-0.84). Among current tobacco users, e-cigarette experimentation was not found to be associated with changes in support for any tobacco control policies.

Among former smokers, support for advertising bans (aOR=1.10, 95% CI: 1.01-1.19), keeping tobacco out of sight (aOR=1.09, 95% CI: 1.00-1.18) and banning online sales (aOR=1.16, 1.07-1.25) increased between 2012 and 2014. Former smokers who had experimented with e-cigarettes were less likely to support any of the tobacco control policies assessed. This included standardized packaging for tobacco (aOR=0.70, 95% CI: 0.60-0.82), banning flavors (aOR=0.72, 95% CI: 0.61-0.84), and keeping tobacco out of sight (aOR=0.77, 0.65-0.90).

## DISCUSSION

This analysis of recent data for the EU Member States, representing a population of 508 million people, has shown that experimentation of former-smokers and non-smokers with e-cigarettes is associated with reduced levels of support for a wide variety of tobacco control policies.

E-cigarette experimentation was inversely associated with support for a range of tobacco control policies including: banning advertising, banning tobacco displays, online sales, flavors, standardized packaging, tax increases and actions to reduce illicit trade. These associations potentially validate concerns that e-cigarette experimentation among former smokers and non-smokers may undermine tobacco control efforts^10^. This represents a potentially large population-health impact that extends beyond the debate about harm reduction, risks and benefits of actual use of e-cigarettes. The association between e-cigarette use and lack of support for tobacco control policies may also have an element of reverse causality, which cannot be excluded due to the cross-sectional design of this study. Notably, previous research has indicated that a large proportion of people who use e-cigarettes do so to circumvent smoking bans, which might indicate a pre-existing negative view of tobacco control efforts^2^.

At a population level, we identified modest changes in overall levels of support for the tobacco control policies examined between 2012 and 2014, the timeframe significantly before the implementation of the TPD. It will be of interest to see how these factors are altered after the TPD is implemented in the 28 EU MS. Advertising bans, placing tobacco products out of sight and bans of online sales were more likely to be supported in 2014 compared to 2012 among former and never smokers. Among current smokers, however, there were no increases in support for any of the policies examined. This suggests that campaigns will need to be able to reach smokers if tobacco control policies are to be furthered, as support from both non-smokers and smokers is essential for the successful implementation of such policies^15^. The association of e-cigarette experimentation with reduced support for standardized packaging amongst non-smokers is also noteworthy.

### Limitations and strengths

There are some limitations to this work that should be borne in mind. Although a variety of sociodemographic factors, views and use of tobacco/ecigarettes were included, it is likely that there might be other factors that influence population support for tobacco control policies. The Eurobarometer survey includes independent samples each year, thus analyses of change do not represent longitudinal individual-level change, which would potentially be more informative. As these data were cross-sectional, causal associations cannot be made, warranting prospective cohort studies to monitor TPD implementation to assess changes in support for tobacco control policies and the effects of the EU TPD^16^.

Despite its limitations due to the available data, this study provides important information using a large and representative sample of the EU population; it allows for analyses that control for several confounding factors and yields results that are generalizable at the EU level. Data were collected on several tobacco control policies and we could explore nuances in respondents’ opinion on different aspects of tobacco control. Additionally while there has been some research on the issue of e-cigarette regulation, lack of data among never smokers has been noted by previous work^17^.

## CONCLUSIONS

While support for tobacco control policies in Europe is high, experimentation with e-cigarettes among former smokers and never smokers was consistently associated with reduced support for a variety of tobacco control interventions, including standardized packaging, taxation and banning of tobacco advertising. The implications of this for future tobacco control policies are unknown, but these data support concerns that experimentation with e-cigarettes may be affecting public support for tobacco control policies within certain sections of the population.
